# Small-Molecule Ligands of Rhodopsin and Their Therapeutic Potential in Retina Degeneration

**DOI:** 10.3390/ijms26188964

**Published:** 2025-09-15

**Authors:** Zaiddodine Pashandi, Beata Jastrzebska

**Affiliations:** 1 Department of Pharmacology, School of Medicine, Case Western Reserve University, 10900 Euclid Ave., Cleveland, OH 44106, USA; zxp144@case.edu; 2 Cleveland Center for Membrane and Structural Biology, School of Medicine, Case Western Reserve University, 10900 Euclid Ave., Cleveland, OH 44106, USA

**Keywords:** ligands of rhodopsin, misfolded rod opsin, retinitis pigmentosa, degenerative disorders, therapeutic potential

## Abstract

Rhodopsin, the prototypical Class A G protein-coupled receptor (GPCR) and visual pigment of rod photoreceptors, has long served as a structural and mechanistic model for GPCR biology. Mutations in rhodopsin are the leading cause of autosomal dominant retinitis pigmentosa (adRP), making this receptor a critical therapeutic target. In this review, we summarize the chemical, structural, and biophysical features of small-molecule modulators of this receptor, spanning both classical retinoid analogs and emerging non-retinoid scaffolds. These ligands reveal recurrent binding modes within the orthosteric chromophore pocket as well as peripheral allosteric and bitopic sites, where they mediate folding, rescue trafficking, photocycle modulation, and mutant stabilization. We organize ligand performance into a three-tier framework linking binding affinity, cellular rescue potency, and stability gains. Chemotypes in tier 2, which show sub-micromolar to low-micromolar activity with broad mutant coverage, emerge as promising candidates for optimization into next-generation scaffolds. Across scaffolds, a recurring minimal pharmacophore is evident by a contiguous hydrophobic π-surface anchored in the β-ionone region, coupled with a strategically oriented polar handle that modulates the Lys296/Glu113 microenvironment, offering tractable design vectors for non-retinoid chemotypes. Beyond the chromophore binding pocket, we highlight opportunities to exploit extracellular loop epitopes, cytoplasmic microswitch clefts, dimer/membrane interfaces, and ion co-binding sites to engineer safer, state-biased control with fewer photochemical liabilities. By integrating rhodopsin photobiophysics with environment-aware, multi-state medicinal chemistry, and by addressing current translational challenges in drug delivery, this review outlines a rational framework for advancing rhodopsin-targeted therapeutics toward clinically credible interventions for RP and related retinal degenerations.

## 1. Introduction

### 1.1. Rhodopsin in the Classification of G Protein-Coupled Receptors

G protein-coupled receptors (GPCRs) comprise a superfamily of over 800 membrane proteins in the human genome, representing approximately 4% of protein-coding genes [1]. They mediate physiological responses to a vast array of extracellular signals, including neurotransmitters, hormones, peptides, lipids, and photons. GPCRs are structurally unified by their seven-transmembrane (7TM) domain architecture and are classified into several major classes such as Class A (Rhodopsin), Class B1 (Secretin), Class B2 (Adhesion), Class C (Glutamate), Class D1 (Ste2-like fungal pheromone), Class F (Frizzled), Class O1 (Fish-like odorant), Class O2 (Tetrapod-specific odorant), and Class T2 (Taste) (Table 1), with Class A accounting for over 85% of all GPCRs [2,3].

Rhodopsin (encoded by the *RHO* gene) is the prototypical and most extensively characterized member of Class A GPCRs at the molecular level. Rhodopsin was the first GPCR for which a high-resolution structure was solved at 2.8 Å resolution in 2000 [5], making a turning point in the field of structural biology of membrane proteins. Rhodopsin shares structural motifs with other Class A GPCRs, such as the conserved E/DRY motif at the cytoplasmic end of transmembrane helix 3 (TM3) and the NPxxY motif on TM7 [5,6], but it also exhibits unique features essential for photon detection. Evolutionarily, rhodopsin belongs to the opsin subfamily and shares ancestry with olfactory and melanopsin receptors [7].

### 1.2. Biochemical and Biophysical Similarities and Distinctions of Rhodopsin Compared to Other GPCRs

While rhodopsin conforms to the 7TM topology of Class A GPCRs, its biochemical and biophysical properties set it apart. One of the most distinguishing features is its native ligand, 11-*cis*-retinal, covalently attached to Lys296 located in the TM7 of opsin protein via a protonated Schiff base linkage, forming rhodopsin. 11-*cis*-Retinal acts as an inverse agonist in the dark, maintaining the receptor in an inactive conformation [8]. Photon absorption causes ultrafast (femtosecond scale) isomerization of 11-*cis*-retinal to all-*trans*-retinal, initiating receptor activation [9,10]. Unlike most GPCRs that exhibit basal activity, rhodopsin is nearly completely inactive in its dark state, with basal activity at least 100-fold lower than other GPCRs like, for example, the β2-adrenergic receptor [11]. Its exceptionally high thermal and conformational stability has facilitated crystallization and time-resolved spectroscopy studies, establishing rhodopsin as a model system for studying GPCR activation mechanisms [12,13]. Rhodopsin also possesses specific post-translational modifications, including palmitoylation of Cys322 and Cys323 at the C-terminal tail, N-linked glycosylation at Asn2 and Asn15, and a conserved disulfide bridge between Cys110 and Cys187, critical for proper folding and photoreceptor disk targeting. These features influence its membrane localization, signal transduction efficiency, and resistance to misfolding, which is not uniformly shared with other GPCRs.

### 1.3. Functional Mechanism of Rhodopsin and Its Role as a GPCR Model System

**Figure 1:**
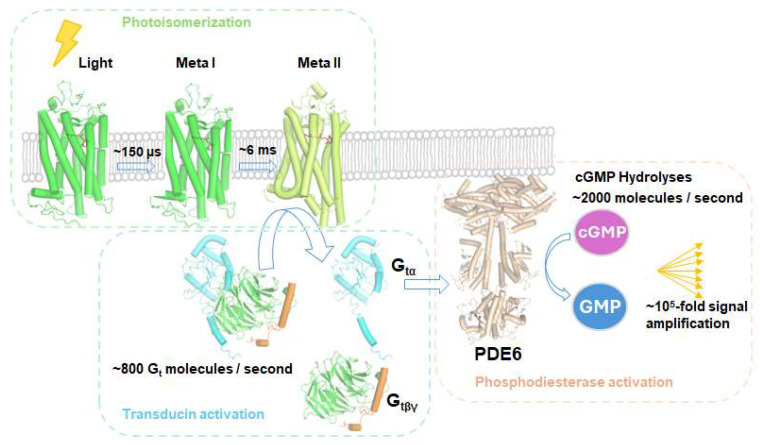
Rhodopsin signaling pathway. Photoisomerization: illumination induces 11-*cis*-retinal within rhodopsin to isomerize, driving the receptor from its ground state through Meta I into the active Meta II conformation. Transducin activation: active Meta II binds the heterotrimeric G protein transducin, promoting GDP-GTP exchange on G_tα_. This leads to dissociation of Meta II-G_t_ complex, and dissociation of G_tα_ from G_tβγ_ dimer. Phosphodiesterase activation: the activated G_tα_ stimulates phosphodiesterase 6 (PDE6), which hydrolyses cGMP to GMP, lowering intracellular cGMP levels and initiating closure of cGMP-gated ion channel in photoreceptor cells.

Functionally, rhodopsin serves as a light-activated molecular switch in rod photoreceptor cells. It operates with exceptional sensitivity, with a single photon capable of triggering a measurable electrical response [14]. Upon photon absorption, photoisomerization of 11-*cis*-retinal to all-*trans*-retinal occurs under 200 femtoseconds, with the primary photoproduct Bathorhodopsin forming within 200 femtoseconds–1 picosecond [15]. The receptor then transitions through intermediates such as Lumirhodopsin and Meta I before reaching the Meta II state. The Meta II state, which persists for approximately 1 min under physiological conditions, represents the catalytically active form that interacts with the heterotrimeric G protein transducin (G_t_) [16]. One activated rhodopsin can activate about 800 transducin molecules per second, and each G_tα_ activates a single cGMP phosphodiesterase, resulting in the hydrolysis of approximately 2000 cGMP molecules per second (Figure 1) [17,18]. This cascade amplifies the response to a single photon by up to 10^5^-fold during phototransduction in photoreceptor cells.

Rhodopsin interacts with various proteins during its functional cycle, including transducin, initiating signal transduction, rhodopsin kinase (GRK1), which phosphorylates activated rhodopsin at multiple sites (Ser334, Ser338, Ser343) to initiate deactivation, and arrestin-1, which terminates G-protein signaling [15,19]. Consequently, during this rhodopsin activation cascade the Schiff base linkage between opsin protein and all-*trans*-retinal is hydrolyzed releasing free retinal. This retinal is recycled through the visual cycle, a complex enzymatic pathway in the retinal pigment epithelium (RPE) cells, involving retinol dehydrogenases (RDHs), lecithin retinol acyltransferase (LRAT), and retinal pigment epithelium-specific 65 kDa protein (RPE65) isomerohydrolase, which catalyzes the rate limiting conversion step. The complete 11-*cis*-retinal regeneration requires approximately 10–15 min. Disruption of this cycle impairs chromophore availability, delays dark adaptation, and contributes to progressive retinal degeneration [20].

### 1.4. Biological and Clinical Importance of Rhodopsin

Biologically, rhodopsin constitutes more than 90% of the protein within outer segment disks of rod photoreceptor cells, with each cell containing ~3 × 10^7^ rhodopsin molecules [21]. Approximately 700–1000 rhodopsin molecules are embedded per disk, with ~1000 disks per rod outer segment. Rhodopsin is synthesized in the inner segment, trafficked via the connecting cilium, and inserted into newly formed disks at the base of the outer segment [22]. Disk shedding and renewal occurs daily, with about 10% of disks being phagocytosed by the RPE cells each morning [23]. The half-life of rhodopsin under physiological conditions is approximately 10 days [24]. Proper regulation of rhodopsin biosynthesis, folding, and trafficking is essential for photoreceptor homeostasis. Disruption of rhodopsin homeostasis, through overexpression, misfolding, or defective degradation leads to photoreceptor cell death via endoplasmic reticulum (ER) stress, proteasomal overload, and apoptosis [22,25]. The molecular mechanism underlying retinal degeneration caused by rhodopsin misfolding is discussed in detail in other reviews [26,27,28]. For example, the most common P23H mutation in rhodopsin leads to protein aggregation and ER retention, contributing to rod photoreceptor degeneration in RP.

**Figure 2:**
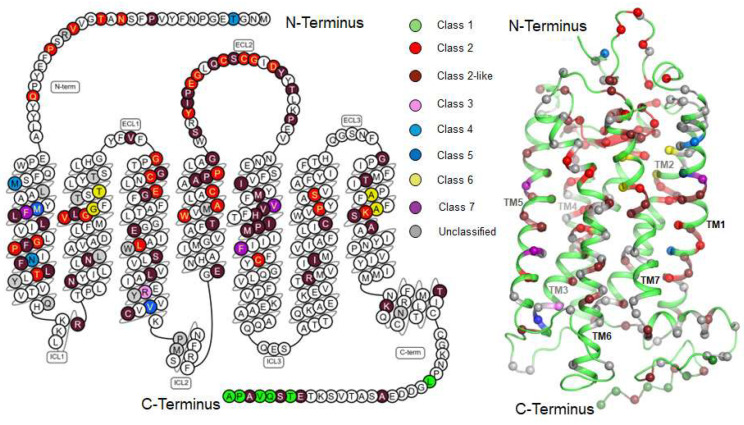
Two- and three-dimensional models of rhodopsin. Clinically common and/or well-studied disease-associated rhodopsin mutations are mapped to their sequence positions and structural locations. Mutation classes are distinguished by color coding: Class 1 (green); Class 2 (red); Class 2-like (dark red); Class 3 (pink); Class 4 (light blue); Class 5 (dark blue); Class 6 (yellow); Class 7 (purple); and unclassified (gray). This color coding corresponds to both 2D and 3D models. The mutations shown in the figure are representative mutations covering the main types of Class 1–7.

Clinically, mutations in the *RHO* gene account for approximately 25–30% of all adRP cases, affecting more than 1 in 4000 individuals in the U.S. and 1 in 5000 globally [29,30]. Over 150 pathogenic mutations have been reported, and they are classified into several functional categories: Class 1, properly folded and trafficked, but functionally impaired (e.g., L328P, Q344R); Class 2, misfolded and retained in the ER (e.g., P23H, T17M); Class 3, disrupted vesicular traffic and endocytosis (e.g., R135G/L/P/W); Class 4, altered post-translational modifications and reduced stability (e.g., T4K, M39R); Class 5, altered transducin activation (e.g., M44T, V137M); Class 6, constitutively active or impaired G-protein activation (e.g., K296E, G90D); and Class 7, dimerization deficiency (e.g., F45L, F220C), (Figure 2) [27,31] Most rhodopsin mutations remained unclassified until the recent application of deep mutational scanning , which revealed that the majority of known variants cause misfolding or partial misfolding and can be categorized as Class 2 mutations (Figure 2, Class 2-like) [31,32].

Therapeutic strategies for rhodopsin-related retinal disorders are under active development and include (i) Gene therapy with AAV-mediated delivery of wild-type *RHO* or gene silencing combined with replacement [33]; (ii) Pharmacological chaperone small molecules, including analogs of 11-*cis*-retinal or non-retinoid stabilizers to promote proper folding and trafficking [32,34,35,36,37,38]; (iii) Pharmacological degraders enhancing the degradation of misfolded rhodopsin [39]; (iv) Neuroprotectants preserving the viability and function of photoreceptors through other means, such as reducing ER stress or improving energy metabolism [40]; (v) RNA-based therapies, antisense oligonucleotides, and RNA interference to modulate mutant transcript levels [41]; (vi) Stem cell therapy, utilizing generation of retinal cells from induced pluripotent stem cells (iPSCs) to replace degenerated photoreceptors [42]; and (vii) CRISPR/Cas9 genome editing, correcting specific *RHO* mutations [43].

Thus, rhodopsin not only plays a central physiological role in vision but also serves as a crucial target for therapeutic interventions and as a paradigm for understanding GPCR-related disease mechanisms. Here in this review, we provide an overview of small-molecule ligands that can directly bind to rhodopsin and/or ligand-free opsin and potentially serve as a pharmacological chaperone to improve disease conditions associated with this receptor misfolding. We followed a systematic review approach to find all related evidence applicable for this review. We performed a search within the PubMed database using a combination of keywords: (“rhodopsin”, “retinal protein”, “opsin”, and “visual pigment”) as well as (“ligand”, “agonist”, “antagonist”, “modulator”, “allosteric”, “binding”, “small molecule”, “chromophore”, “analogs,” “chaperones”, “derivatives”, “pharmacological”, and “compounds”), which resulted in 6715 papers. From this list, we selected 40 papers using the Rayyan online tool considering the following eligibility criteria: (i) being original papers, (ii) papers only focused on mammalian rod opsin, and (iii) papers that include any small molecules, which directly interact with rod opsin. In addition, (iv) we excluded papers with endogenous ligand or retinal analogs that exclusively focus on rhodopsin photochemistry without any therapeutic relevance.

## 2. A Comprehensive Overview of Therapeutic Small-Molecule Ligands of Rhodopsin

Based on the reviewed literature, ligands binding to rhodopsin can be broadly divided into two categories. The first category includes ligands with pharmacological effects on rhodopsin/rod opsin structure and stability, referred here as therapeutic ligands (Table 2). The second category comprises ligands designed primarily to probe the mechanisms of pigment photoisomerization, which we term photochemical ligands. In this section, we focus on therapeutic ligands, organizing them according to their distinct properties, while photochemical ligands are discussed in the following section.

### 2.1. Therapeutic Ligands Based on Core Scaffold Features and the Conformational States They Stabilize

**Figure 3:**
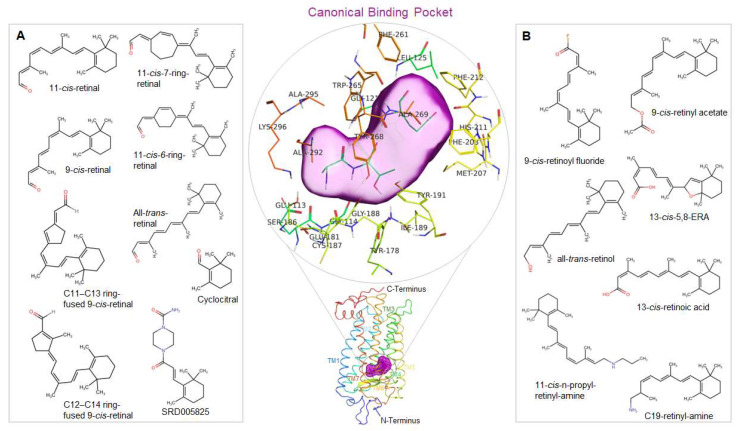
Rhodopsin’s orthosteric site. The canonical chromophore binding pocket and its key residues (middle panel). Box (**A**) shows retinoid ligands containing aldehyde groups capable of forming a Schiff base linkage with Lys296. Box (**B**) presents retinoid-mimic ligands in which the aldehyde has been substituted with alternate functional groups. Despite these differences, both classes of ligands can interact with opsin within the canonical binding pocket.

**Figure 4:**
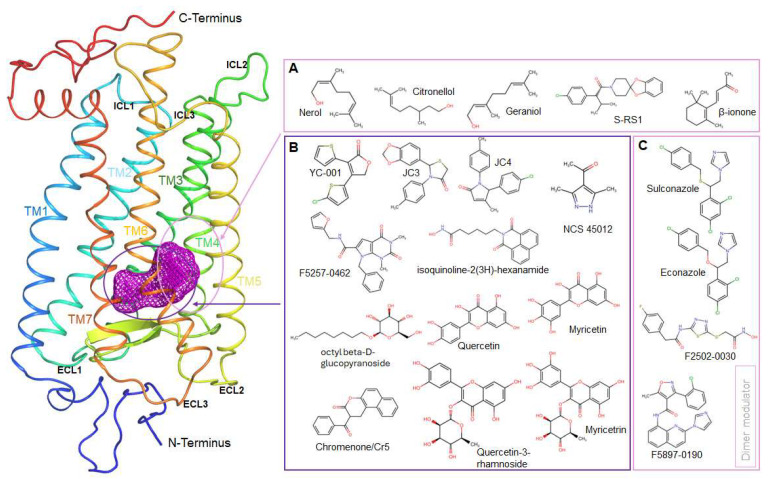
Representation of non-retinoid ligand that can occupy rhodopsin’s orthosteric binding pocket. Box (**A**) and Box (**C**) depict non-retinoid ligands that primarily occupy the β-ionone cavity, engaging grooves formed by TM3, TM5, and TM6. Box (**B**) illustrates non-retinoid ligands that penetrate more deeply into the orthosteric site, approaching TM7 but without forming a Schiff base linkage.

Nearly 80% of all reported ligands represent ligands that bind to canonical orthosteric binding pocket (Figure 3 and Figure 4). This class of compounds include classical retinoids and their analogs (e.g., 11-*cis*-retinal [44], 9-*cis*-retinal [45], β-ionone [45], locked ring-retinal [34,47], 9-*cis*-retinyl acetate [48], retinyl amines [51], and retinoyl fluorides [50]), which share a β-ionone (or isosteric bicyclic) hydrophobic anchor linked to a conjugated (CH=CH)n chain that terminates in an aldehyde or latent imine precursor. This polar group orients toward Lys296/Glu113 to form or mimic the protonated Schiff base, thereby stabilizing the dark state while retaining photoisomerizability.

Retinoid-derived compounds (e.g., 13-*cis*-5,8-ERA [55] and retinobenzaldehydes [56]) fall within the same category, as do newer non-retinoid hydrophobic chaperones (e.g., YC-001 [59], chromenones [36], SRD005825 [57], pyrazol-ketone NSC 45012 [38], and JC3/JC4 [32]). These scaffolds preserve a hydrophobic aromatic/alkenyl anchor that π-stacks with Trp265/Tyr268, together with a polar “warhead” (aldehyde, nitrile, carboxylate, amide, α,β-unsaturated carbonyl, acetate, amine, or halogen) that points toward the Lys296 within the binding pocket or its H-bonding partners (Glu181/Ser186). Functionally, they act mainly as inverse agonist chemical chaperones. In addition, flavonoids such as quercetin and myricetin, when bound in the orthosteric site, fall into this group [67]. Similarly, Spiro-RS analogs can be categorized here, as the chlorophenyl ring of S-RS1 overlaps the β-ionone position of the endogenous ligand [63].

**Figure 5:**
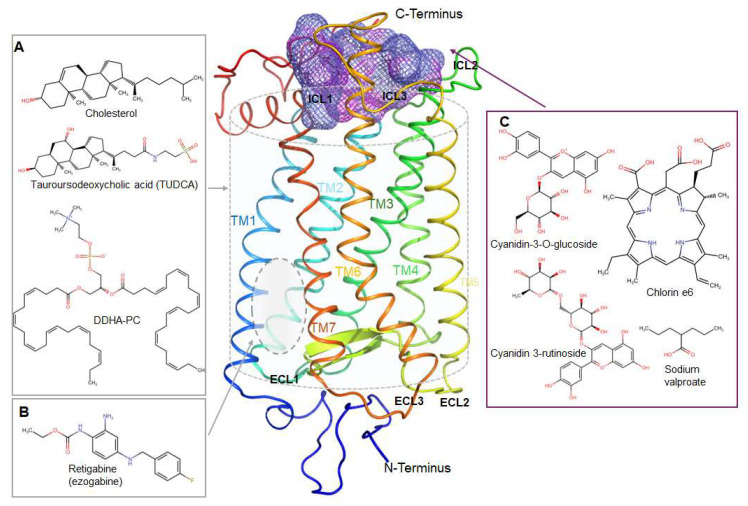
Representation of non-retinoid ligand that interact with rhodopsin at allosteric sites. Box (**A**) illustrates lipidic non-retinoid compounds that interact primarily with transmembrane regions, while Box (**B**) shows non-retinoid ligands engaging the extracellular region of rhodopsin. Box (**C**) shows ligands that interact at cytoplasmic surface.

The remaining ~20% constitute true allosteric modulators: (i) Flavonoids [77], anthocyanins and chlorin/porphyrins [69] (Figure 5). These compounds are rich in multiple hydroxyls or propionic acids, bind extracellular or cytoplasmic clefts, stabilizing or destabilizing Meta II kinetics through H-bond nets; (ii) Lipid-tail scaffolds (cholesterol [73], DHA-PC [72]), which insert into TM1–2 or TM5–6 grooves, modulating helix kinks by long hydrophobic contacts plus a single sterol or phosphocholine polar head [78]; and (iii) Aromatic aminopyridine core (e.g., retigabine [68]), imidazole core, and dimer-interface hits, which combine a hydrophobic multi-ring with an imidazole/thiazole nitrogen that quenches Trp265 and perturbs oligomerization [75].

Functionally, ~75% of the small molecules behave as inverse agonists or dark-state stabilizers, ~20% as agonist/pro-agonist chromophores (e.g., 9-*cis*-retinyl acetate [48] and all-*trans*-retinal [48]), and ~5% as kinetic modulators of Meta I/II or dimer state; irreversible covalent mechanisms are rare (e.g., retinoyl fluorides and C19-retinyl-amine) [50]. Despite diverse scaffolds, the recurring themes are as follows: (i) a contiguous hydrophobic π-surface of ≈12–16 Å that overlays the β-ionone pocket or a lateral cleft (anchoring via van der Waals/π-stacking with Trp265/Phe261/Tyr268 or membrane-embedded residues); (ii) a single strategically oriented H-bond donor/acceptor (aldehyde, carbonyl, amide, nitrile, nitrogen heteroaromatic, hydroxyl, carboxylate) that either forms/neutralizes the Schiff base region (Lys296/Glu113/Glu122/Glu181) or engages cytoplasmic acidic residues (Glu134/Arg135 network) to bias conformational equilibria; and (iii) optional rigidity elements (ring locking, azobenzene, and spiro centers) that reduce entropic penalty and impose conformational bias (dark vs. Meta I/II). Thus, despite broad scaffold diversity, an overarching “hydrophobic + polar handle” minimal pharmacophore underlies ligand control of folding, activation, and signaling trajectory, offering tractable vectors (hydrophobic surface topology, single polar pivot, and conformational constraint) for next-generation non-retinoid therapeutic design.

### 2.2. Therapeutic Ligands Based on Binding Affinity to Rod Opsin and Effects on Maturation and Membrane Trafficking

Three performance tiers emerge when ligands are ranked by binding affinity to rod opsin (K_d_), pharmacological potency for rescuing folding, pigment formation or trafficking (EC_50_), and gain in biochemical stability indices (thermal half-life).

Tier 1. High-affinity chromophore memetic: defined by strong binding (K_d_ ≤ 100 nM), potent functional rescue (EC_50_ ≤ 1 µM), and ≥4-fold increases in mature pigment at the plasma membrane. These are dominated by *cis*-retinoid aldehydes, including 11-*cis*-retinal, 9-*cis*-retinal, and photostable locked retinal analogs. These compounds bind to rod opsin at sub-nanomolar concentrations forming a Schiff base with Lys296. Upon binding to misfolding mutants such as P23H and T17M, they increase opsin maturation to over 60% of wild-type receptor levels [31,44]. Besides the endogenous ligand, 9-*cis*-retinal and notably the ring-locked retinal show the lowest EC_50_ values, uniquely providing protection against chronic cellular stress caused by RP-associated mutations [34].

Tier 2. Pharmacological stabilizers: characterized by moderate affinity (K_d_ ≈ 0.1–5 µM, EC_50_ ≈ 1–50 µM) and 1.5–4-fold functional rescue. This class includes small non-retinoid molecules, amphipathic acids and heteroaryl amides, with notable examples being 13-*cis*-5,8-ERA [55], SRD005825 [57], JC3/JC4 [32], YC-001 [59,60], and chromenone derivatives [36]. Their polar moieties neutralize the Schiff base, promoting a native-like conformation that enhances proper folding and membrane trafficking of misfolded rhodopsin mutants. Among these, 13-*cis*-5,8-ERA, YC-001, chromenone/CR5, F5257-0462, and JC3/JC4 have emerged as leading candidates for therapeutic development, demonstrating sub-micromolar binding, micromolar-range rescue, and a broad mutant coverage. In addition, chromenone/CR5, F5257-0462, and JC3/JC4 provide in vivo photoreceptor protection without relying on reactive aldehyde group.

Tier 3. Mechanistic probes: defined by weak binding affinity (K_d_ > 5 µM), non-covalent or equilibrium interactions, and often undefined or high EC_50_ values (>50–100 µM), with limited functional rescue (<1.5-fold). Include non-retinoid ligands at allosteric positions, such as retigabine [68], chlorin e6 (Ce6) [69,70], cyanidin-3-glucoside (C3G) [65] and lipidic compounds [72,73]. These agents are effective at modulating Meta II signaling but confer only modest thermal stability gains (ΔT_m_ ≤ +1 °C), serving primarily as structural and mechanistic tools. Rather than acting as pharmacological chaperones, they function as reversible actuators: a light-driven *cis* → *trans* isomerization that triggers Meta II activation with sub-micromolar precision.

In practical terms, ligands that combine sub-micromolar EC_50_ with >4-fold improvement in folding and trafficking and ≥2 °C boost in thermal stability in Tier 1 define the “drug-ready” space, currently satisfied only by *cis*-retinoids and selected ring-locked analogs, while Tier 2 scaffolds offer chemically tractable leads for optimization toward those benchmarks. Tier 2 contains most of the non-retinoid ligands which rescue folding and trafficking of multiple Class 2 mutants and Class 2-like, including the most common P23H, T17M, G106R, D190N and P267L, driving up to 4-fold increase in properly matured pigment and suppress ER-stress signaling in cell models. Several compounds in this tier show improved retinal morphology and function in vivo in animal models of retinitis pigmentosa without inducing phototoxicity or retinal lipid accumulation.

### 2.3. Mapping Binding Location and Interaction of Therapeutic Ligand with Rod Opsin

**Figure 6:**
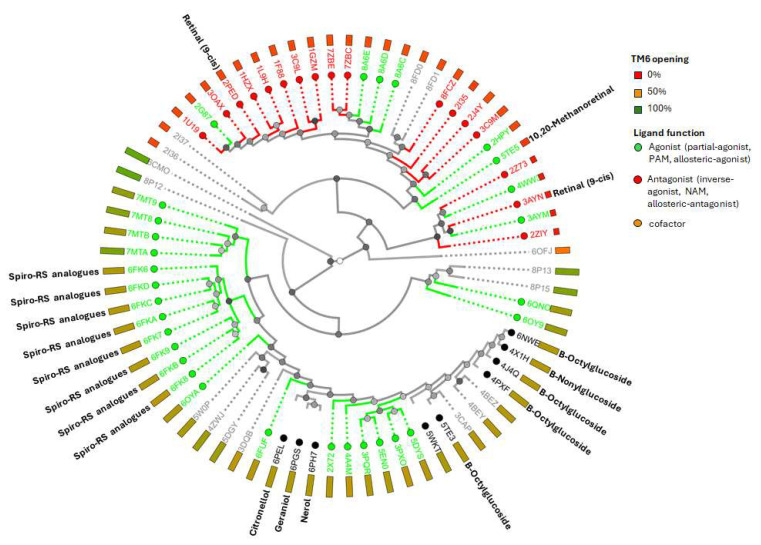
Available PDBs of rod opsin protein structures. The degree of TM6 opening (percent of active conformational state) is indicated by colored rectangular boxes. PDB IDs containing different types of compound or conjugated molecules are marked by colored circular boxes according to ligand function. For structures that include small-molecule ligands, the ligand names are shown above the corresponding PDB ID.

To date, among the 73 PDB entries for the opsin receptor identified through the RCSB and GPCRdb databases (Figure 6), 63 correspond to bovine rod opsin. The exceptions are human entries 6CMO, 5W0P, 5DGY, and 4ZWJ, as well as Japanese flying squid entries 4WW3, 3AYM, 3AYN, 2Z73, and 2ZIY. Besides the endogenous ligand (in either *cis* or *trans* conformation), structurally determined ligands to date include S-RS1 and its derivatives (6FKD, 6FK7, 6FK8, 6FKC, 6FKA, 6FKB, 6FK9, 6FK6), 9-*cis*-retinal (2PED, 3AYN), nerol (6PH7), geraniol (6PGS), citronellol (6PEL), several non-ionic detergents (6NWE, 5WKT, 5TE3, 4X1H, 4PXF, 4J4Q), and 10,20-methanoretinal (11-*cis*-6-membered-ring-retinal or locked-retinal) (5TE5) [79]. However, except for 9-*cis*-retinal [80], 10,20-methanoretinal [79], and S-RS1 derivatives [63], the biophysical and biochemical effects of the other compounds on rod opsin have not yet been studied. Therefore, here a combination of experimental and computational data was used to map the ligand-binding locations.

Therapeutic ligands of rod opsin generally adopt a limited number of binding topographies, reusing a small set of interaction motifs (Figure 3, Figure 4 and Figure 5). Most compounds either occupy the orthosteric chromophore pocket around Lys296 or engage shallower allosteric sites, such as clefts on the extracellular or cytoplasmic surfaces near charged “hinge” residues (Lys134/Arg135) or the acidic cluster of ECL2. A smaller subset instead targets protein–protein or lipid-interaction surfaces, including dimer interfaces, G protein-binding regions, and cholesterol-associated grooves.

A more detailed description of these binding modes is provided below.

(I). The canonical or orthosteric chromophore binding pocket of rhodopsin, located deep between transmembrane helices TM3, TM5, TM6, and TM7, accommodates a diverse range of ligands including retinoids analogs (9-*cis*-retinal), non-retinoid compounds (YC-001, SRD005825, CR1–CR5, JC3/JC4), and covalent inhibitors such as retinyl-amine and retinoyl fluoride. Weak competitors like β-ionone and NSC 45012 also target this site. Ligand recognition is anchored by several key residues: (i) Lys296 (TM7), forms a covalent protonated Schiff base with retinoid aldehyde or engages in electrostatic hydrogen-bond interactions with non-aldehyde analogs; (ii) Glu113 (TM3), serves as the counter-ion to the Schiff base and helps shape the local electrostatics; and (iii) a hydrogen bonding network comprising Glu181, Tyr191, Tyr192, and Ser186 at the ECL2/TM5 interface, frequently engages with polar functional groups. Additionally, (iv) hydrophobic and aromatic (π–π) contacts with Trp265, Tyr268, Phe212, Ala117, Thr118, and Ala292 cradle β-ionone-like or aromatic scaffolds, acting as a hydrophobic anchor, whereas Glu122 (TM3) occasionally accepts H-bonds (e.g., from RS1 carbonyls, nerol, citronellol, and geraniol). Together, these residues enable diverse interaction types: covalent attachment (Schiff base or irreversible amide), hydrogen bonds and salt bridges with acidic side chains, van der Waals and π–π stacking, and water-mediated networks. Functionally, most orthosteric ligands act as inverse agonists or chemical chaperones, stabilizing the dark state, slowing Meta II transition kinetics, and rescuing folding/trafficking defects of Class 2 opsin mutants. Some compounds, such as 9-*cis*-retinyl acetate prodrug, can regenerate pigment and function as pro-agonist, while covalent agents like acyl fluorides or retinyl-amines can irreversibly inactivate constitutively active mutants.
(II). The “β-ionone surface cleft”, also known as the peri-orthosteric pocket, is located near the outer leaflet of the membrane, spanning TM5, TM6, and ECL3. This shallow, hydrophobic site accommodates ligands such as the “stabilizers” reported by Pasqualetto et al. [62] and spiro ring elements of RS ligands during channel-like conformational openings [63]. Key residues forming this cleft include the Phe283–Ile290 belt at the TM6 tip and ECL3, notably Pro285, Ile286, Phe287, and Met288, with occasional hydrogen bonding to Asp282. Ligand interactions are predominantly characterized by shallow hydrophobic packing and, in some cases, a single polar contact. Rather than acting as direct competitors at the orthosteric site, these ligands typically function as allosteric facilitators, enhancing retinal binding kinetics and accelerating pigment regeneration without displacing the native chromophore.
(III). The extracellular ECL2/N-terminus plug acts as a regulatory gate positioned above the orthosteric pocket, involving ECL2 and the N-terminal region [81]. Although no ligands are known to bind exclusively to this site, partial engagement has been observed with flavonoid or econazole [75], which promote an intradiscal channel-like conformation. Key residues include the acidic ECL2 stretch (Pro194–His195–Glu196–Glu197), Glu201 at the TM5 cap, Tyr191, and glycan-bearing residues Asn2 and Asn15 from the N-terminus. Occupation of this site functionally locks ECL2 over the binding pocket, biasing rhodopsin toward Lumi/Meta I or Meta III states and restricting the outward motion of TM5 and TM6. This negative allosteric modulation stabilizes the receptor dark state and can influence both signaling dynamics and folding efficiency in disease-associated rod opsin variants.
(IV). The cytoplasmic clefts of rhodopsin formed around TM1/2/3 and TM2/6/7 near the ICL2 and ICL3 loops serve as regulatory allosteric sites accessible from the intracellular side. These regions accommodate ligands such as Ce6 [70], anthocyanin C3G [65], and valproate [71]. Key residues involved include Glu134 and Arg135 in the conserved E/DRY motif; surrounding hydrophobics such as Ile133, Val138, Leu72, and Phe146; and deeper contacts with Asn302, Thr58, and Met317 near the base of TM7. Ligand interactions include salt bridges and hydrogen bonds to Lys/Arg residues and hydrophobic packing parallel to the membrane plane. These interactions often result in weak (micromolar) bindings that subtly modulate global conformational dynamics, either stabilizing the dark state (as seen with Ce6) or modestly destabilizing Meta II in mutant contexts (high concentrations of C3G or valproic acid).
(V). The dimer and oligomer interfaces, along with membrane-facing grooves, represent peripheral allosteric sites that modulate rhodopsin assembly and signaling. This can include dimer modulator compounds, such as econazole compounds [75], and lipidic molecules, such as cholesterol [73,78], as well as retigabine [68]. For dimer modulator ligands, direct contacts have not been resolved. However, their ability to quench Trp265 fluorescence suggests an allosteric binding mode near the β-ionone pocket but far from Lys296 [75,76]. Econazole ligands likely interact with the dimer at the TM1/TM7–8 interface, the TM3/TM5 or TM5/TM6 rim, and helix 8. While cholesterol binds at multiple grooves, including the TM2–TM3 rim, the TM1–TM2–TM4 cleft, and near the TM7 cap [73,78]. Through its 3β-hydroxyl group, cholesterol forms hydrogen bonds with tyrosine and threonine residues, while its sterol ring system engages in dense hydrophobic packing that influences transmembrane helix kinks. In addition, cholesterol stabilizes the inactive state ensemble by tightening helical packing and reinforcing the NPxxY/H8 motif, modulating photoreceptor responsiveness and long-term structural integrity [73]. Functionally, both these ligands can alter oligomerization dynamics, slow Meta II decay, and shift ERG response kinetics.
(VI). Irreversible active-site-directed chemistries target the orthosteric lysine residue (Lys296) using electrophilic or nucleophilic ligands such as retinoyl fluorides (–COF) and retinyl-amines (–NH_2_). These compounds form stable amid or iminium linkages with Lys296 within the orthosteric site, resulting in non-bleachable pigments with characteristic absorption around λ_max_ ~365 nm [50]. By covalently locking the chromophore site, these ligands permanently silence constitutively active opsin mutants, blocking unwanted activity and halting pathological signaling, offering a potential therapeutic route for dominant-negative retinal diseases.

Collectively effective therapeutic design for misfolded or dysfunctional opsins largely centers on orthosteric ligands that avoid reactive aldehydes to minimize phototoxicity and off-target effects. Successful chemotypes include butyrolactones (JC3/JC4), chromenones (CR5), spiro-butyrones (RS2), and polar nitrile or amid derivatives. These compounds typically combine a hydrophobic ring that mimics the β-ionone moiety for anchoring deep within the pocket, with a polar headgroup capable of forming stabilizing hydrogen bonds within the Lys296, Glu181, and Tyr191 cluster, reinforcing the dark-state conformation. While allosteric rescue is possible through regions such as the ECL2 clamp or cytoplasmic clefts, these effects are often modest, micromolar in potency, and context-dependent, varying with chromophore state or pH. Modulating dimerization or oligomerization presents a distinct regulatory lever that may impact receptor kinetics and signaling sensitivity, though it remains unproven for folding correction. Lastly, irreversible covalent strategies such as those with retinoyl fluorides or retinyl-amines can silence hyperactive mutants by permanently locking the active site, but due to the risk of off-target toxicity, they are better suited for mechanistic studies than for therapeutic application.

### 2.4. Ligands with Counter-Intuitive or Mixed Functional Profiles

A subset of ligands in the current dataset exhibits functional profiles that appear contradictory at first glance. These behaviors typically arise from binding site location, ligand chemistry, receptor genotype, and experimental context and should be annotated explicitly to avoid misinterpretation.

#### 2.4.1. Ligands That Shorten Meta II Lifetime or Fail to Rescue Folding Defects

(i). Sodium valproate, a weak intracellular-cleft binder with modest stabilization of the wild-type dark state, yet markedly reduced Meta II half-life in the I307N mutant (16.3 → 5.2 min) and offered no rescue of that mutant’s dark stability [71]. This suggests a cytoplasmic binding site near TM2/TM7 that perturbs the NPxxY/H8 microdomain, biasing the active-state landscape unfavorably. Therefore, sodium valproate is a poor chemical chaperone for Class 2 rod opsin mutans and can destabilize activation equilibria.
(ii). Retigabine produces pronounced thermal stabilization and improves chromophore regeneration, yet it accelerates Meta II decay (half-time reduced by ~50%). This is consistent with negative allosteric modulation of activation. Retigabine stabilizes the dark state via a TM1/TM7 cleft but disfavors the active Meta II conformation once formed [68].
(iii). Rhodopsin dimer modulators such as econazole series [75]. The R-econazole enantiomer traps Meta III (λ_max_ ≈ 465 nm), fully quenches Trp265 fluorescence, and slows rod photo-response kinetics ex vivo, whereas the S-form primarily reduces sensitivity without kinetic slowing. These stereospecific effects are attributable to dimer/interface allostery rather than orthosteric stabilization and therefore fall outside the classical definition of “chemical chaperones”. Taken together, these distinct binding profiles highlight an unresolved therapeutic question: can such modulators, despite their unconventional mechanisms, be harnessed to counteract constitutively active rod opsin variants?

#### 2.4.2. Dual Orthosteric-Allosteric Behavior in Flavonoids

(i). Flavonoids. Docking and experimental data indicate that aglycone such as quercetin and myricetin can occupy either the rod opsin orthosteric pocket or external sites (ECL2/TM5–TM6), with binding preference modulated by the concentration, protonation state, and chromophore form (11-*cis* vs. 9-*cis*-retinal) [67,77]. Functionally, quercetin increases thermal stability and accelerates regeneration of opsin with 9-*cis*-retinal (chaperone-like effect). It modulates Meta II decay bidirectionally depending on dose and pigment context and promotes self-association (↑BRET), an effect absent in typical orthosteric inverse agonists. These outcomes reflect site promiscuity combined with pH-dependent chemistry of the flavylium core. Glycosides, being bulkier, cannot enter the orthosteric site and primarily act externally.
(ii). Cyanidin-3-glucoside (C3G). At pH 6, increases regeneration rate (for ~65%) but reduces thermal and retinal-release stability (half-time 27.7 min → 10.5 min), and dampens transducin activation [65]. The most likely explanation is its binding to cytoplasmic side that facilitates chromophore entry while slightly destabilizing the pigment core, producing apparently contradictory kinetic and stability readouts.

#### 2.4.3. Photosensitizers and Cytoplasmic Allosteric Ligands That Bias State Equilibria

A representative example for such ligands is Ce6, a weak cytoplasmic-face binder [69,70]. Terahertz spectroscopy shows that Ce6 preferentially stabilizes dark-state global conformational ensemble, while concurrently destabilizes active Meta II state. In vivo, its functional effects are largely attributed to its role as a red-light-harvesting photosensitizer rather than direct engagement with the retinal binding pocket. These modulatory effects occur at micromolar concentrations and are qualitative in nature, lacking well-defined dissociation constants (K_d_) or conventional binding parameters.

#### 2.4.4. Membrane Context as a Determinant of Apparent Paradox

Bilayer cholesterol can stabilize the dark state by reshaping TM helices and NPxxY/H8 geometry without engaging Lys296 [73]. This explains why certain ligands appear stabilizing in membranes but neutral or different in detergent micelles [72]. Thus, assays in detergent, reconstituted membranes, and live cells should be interpreted as distinct regimes.

#### 2.4.5. Mechanistic Basis for Unexpected Outcomes

Several factors contribute to the apparently paradoxical effects of ligands on rod opsin:

(i). State bias determined by binding site. Orthosteric inverse agonists typically stabilize the dark state while often shortening Meta II lifetime. Extracellular allosteric ligands impede TM5/6 motions, suppressing Meta II formation and enriching Meta I/III, whereas cytoplasmic allosteric modulators perturb the NPxxY/H8 microdomain, accelerating active-state decay.
(ii). Chemistry and environmental context. Ligand behavior can depend strongly on chemical form and surroundings. For example, for flavonoids, the receptor protonation state (pH-dependent flavylium vs. quinoidal forms), glycosylation-induced steric bulk, and membrane versus detergent environments alter site accessibility and apparent efficacy. These effects can produce mixed orthosteric and allosteric signatures even within a single chemical scaffold.
(iii). Genotype specificity. Ligand effects can be highly mutation-dependent. A compound that stabilizes P23H may destabilize I307N variant. Sodium valproate exemplifies this, as certain mutants exhibit heightened sensitivity of Meta II to cytoplasmic perturbation. Conclusions must always consider the underlying mutant class (e.g., N-terminal misfolding versus TM7 micro-switch defects).
(iv). Readout artifacts. Some ligands, such as econazole and Ce6, can quench Trp265 fluorescence, artificially distorting Meta II kinetics. Thus, confirmatory measurements using UV/Vis spectroscopy or hydroxylamine reactivity are essential to distinguish true kinetic effects from assay artifacts.

### 2.5. Ligand Strategies for Rescuing Rod Opsin-Associated Inherited Diseases

The literature analysis shows that the most frequently studied rod opsin mutants include P23H, T17M, E181K, G90D/G90V, K296E/M, I307N, and Q344T, with P23H and T17M being the most prevalent. These two mutants serve as canonical Class 2 folding and trafficking benchmarks and are widely used across multiple published studies. Table 3 lists small-molecule ligands with potential to rescue folding and trafficking of misfolding rod opsin mutants.

Our review highlights several recurring patterns.

1. Orthosteric inverse agonists remain the dominant approach for effective rescue of defective rod opsin, with most broad-spectrum correctors targeting the chromophore binding pocket to stabilize the dark state, either covalently (retinoids) or non-covalently (non-retinoids). The chemical class selection is critical for balancing efficacy and safety. Non-retinoid scaffolds, including butyrolactones, chromenones, and RS-series compounds, avoid aldehyde reactivity, enhancing light stability and reducing retinoic acid-related toxicity, while maintaining sub-micromolar affinities. The P23H rod opsin has emerged as the primary “workhorse” mutant for studies evaluation pharmacological properties of these ligands, in both cell and animal models. Promising hits emerging from these studies include YC 001, F5257 0462, CR5, JC3/JC4, quercetin, and SRD005825, as well as hydrophobic modulators like β-ionone. A secondary tier of mutants, including T17M, E181K, G90D/V, K296E/M, and I307N, is commonly used to represent distinct mechanistic classes, such as N-terminal signal sequence defects, ECL2 charge perturbations, constitutive activation, or cytoplasmic misfolding. This creates a key therapeutic trade-off between breadth and specificity: retinoids offer broad rescue potential but are limited by photolability and toxicity, whereas non-retinoids provide narrower target coverage but improved drug-like properties and better tolerability.
2. Allosteric ligands, including flavonoids, econazole stereoisomers, cholesterol, Ce6, and C3G, offer valuable mechanistic insights by modulating Meta II/III kinetics or oligomerization states. However, their effects are often mutant-specific and may be destabilizing in certain contexts, emphasizing the importance of carefully matching ligand strategy to the mutation class.

## 3. Brief Overview of Photochemical Ligands

Although a detailed analysis of photochemical ligands is beyond the primary objectives of this study, our literature review identified 75 distinct small-molecule ligands in this class, comprising 63 retinal/retinol derivatives and 12 non-retinal chromophore mimics such as β-ionone, chain-truncated aldehydes, Schiff base surrogates, and ring-truncated polyenes, which we summarized in this paragraph. These findings may help rationalize and extend the trends cataloged in the previous section.

Across these analogs, rhodopsin structure–function modulation converges on a limited set of “control knobs” (Table 4). These control knobs illustrate how modifications of photochemical ligands influence rhodopsin structure and function. From this framework, distinct design rules can be derived for specific objectives, including (i) photostabilizing the dark state by locking the C11=C12 bond in retinal and reinforcing the Trp265 pocket; (ii) shifting the equilibrium toward Meta II (stronger agonism) by lowering the Schiff base pKa without weakening the steric brace; and (iii) generating partial or inverse agonists by deleting retinal C19, removing the ring, or introducing moderate steric bulk at C10/C12. In addition, these principles provide general insights for the rational design of non-retinoid pharmacological chaperones, where most folding-defective Class 2 mutants can be rescued in cultured cells by hydrophobic pocket fillers that (i) insert into the vacant chromophore site, (ii) transiently restore TM2/TM3/TM4 packing to enable glycosylation and ER export, and (iii) can later dissociate, or in some cases be photoreleased, once folding is achieved.

Considering opportunities for non-retinoid pharmacological chaperones, our dataset highlights three promising chemotypes (Table 5). Collectively, the patterns indicate that agonist efficacy in rhodopsin depends less on overall binding affinity and more on precise redistribution of steric strain and Schiff base protonation thermodynamics. Importantly, the data highlights minimal, non-retinoid hydrophobic scaffolds, such as chain-truncated aldehydes, β-ionone, and fluorinated or acyclic fragments, as promising chaperones to stabilize folding, support correct glycosylation, and bias specific conformational ensembles (dark state, Meta I trap, long-lived Meta II, or photocyclic intermediates) for therapeutic or optogenetic applications.

From these observations, a design roadmap emerges for medicinal chemistry: new non-chromophoric scaffolds should (i) mimic the hydrophobic volume of the β-ionone ring and proximal polyene region, (ii) present a hydrogen-bond donor/acceptor near Glu113 to bias Meta I vs. Meta II equilibria, and (iii) incorporate solubilizing handles for systemic delivery. Such leads could stabilize misfolded rod opsin variants during biosynthesis, restore proper N-glycosylation and trafficking, and ultimately provide an urgently needed therapeutic strategy for adRP.

## 4. Development of Therapeutic Ligands for Rod Opsin: Status, Limitations, and Prospects

### 4.1. Current Stage of Small-Molecule Ligand Development

Based on chemical space explored in this review therapeutic efforts have coalesced around four principal ligand classes: (i) Chromophore replacements and pro-agonists (e.g., 9-*cis*-retinal and 9-*cis*-retinyl acetate oral prodrugs) that reconstitute photoactivity but suffer from phototoxicity risks and off-target retinoid receptor engagement; (ii) Reversible non-retinoid orthosteric inverse agonists/chemical chaperones (e.g., YC001, CR1–CR5, JC3/JC4, RS-series, SRD005825), which bind within or adjacent to the retinal pocket, lower basal activity, and stabilize the dark-state fold, frequently rescuing trafficking-defective mutants in cell systems and preserving photoreceptor structure and function in rodent models; (iii) Emerging allosteric and bitopic modulators, small molecules targeting ECL2, the cytoplasmic cleft, or membrane/dimer interfaces (e.g., flavonoids, Ce6, C3G, econazole analogs), and macromolecules such as nanobody Nb2 or G_t_ and β-arrestin-derived peptides (in this review we did not focus on macromolecules). These agents demonstrate that surfaces beyond the canonical chromophore pocket are druggable and can bias downstream signaling or folding; and iv) Irreversible/covalent blockers and tool compounds (retinyl-amines, retinoyl fluorides) that lock key photointermediates or disable chromophore loading, useful mechanistic probes but poor therapeutic candidates.

Therapeutic development targeting rhodopsin has progressed notably beyond the proof-of-concept stage. A subset of orthosteric non-retinoid ligands now demonstrates key preclinical milestones, including sub-micromolar binding affinities, reproducible thermal stabilization (ΔT_m_ of +2–9 °C), broad rescue efficacy across numerous adRP mutants, and functional preservation in vivo, as evidenced by maintained ERG amplitudes and ONL thickness [32,35,36]. In contrast, allosteric chemotypes and biologics largely remain in earlier stages of discovery or lead optimization, with limited characterization of their kinetics, structural binding modes, and safety profiles.

A promising trend is the emergence of state-aware ligand design, leveraging the unique conformational landscape of the rhodopsin photocycle. Medicinal chemistry efforts increasingly target specific structural “locks”, such as the PSB–Glu113 counter-ion network [88,89], the Arg135–Glu247 ionic lock [90], and the NPxxY/H8 clamp [6], or transient pockets formed during activation, such as TM6 outward movement and ECL2 rearrangements [15]. State-selective strategies are exemplified by bitopic small molecules that bridge orthosteric and intracellular domains.

Simultaneously, the role of the membrane environment in modulating rhodopsin activation and ligand efficacy is gaining recognition. Ions (e.g., Na^+^, Zn^2+^, SCN^−^), lipid composition (DHA/PE-rich vs. POPC membranes), and formulation systems (nanodiscs, liposomes), along with buffer variables such as pH and trace metals, are known to influence the Meta II/Meta III equilibrium and rescue outcomes [88,91,92,93,94,95]. However, these factors are still infrequently optimized during early-stage screening, representing an untapped opportunity for improving translational success.

Collectively, the field now rests on a more solid mechanistic and structural foundation. There exists a credible pipeline of chemically diverse, state-specific, and environmentally tunable modulators with demonstrated efficacy in preclinical models. However, clinical translation remains elusive, hindered by challenges in pharmacokinetics, safety profiling, and the need for rigorous validation across heterogeneous patient genotypes. Addressing these gaps will be essential for realizing the therapeutic potential of rhodopsin-targeted interventions.

### 4.2. Limitations and Bottlenecks in Drug Development for Pathogenic Rhodopsin

Despite these advances, several critical challenges continue to hinder clinical translation. First, orthosteric bias remains a major concern. The intense focus on the retinal binding pocket risks interfering with the natural visual cycle, particularly through retinoid-like scaffolds that may form toxic photo adducts or activate off-target nuclear receptors. Furthermore, potency and efficacy metrics across the literature remain inconsistent and poorly standardized. Many studies rely solely on ΔT_m_ shifts or trafficking rescue without reporting fundamental pharmacological parameters, such as K_d_, EC_50_, or residence time. Variation in assay platforms, ranging from detergent micelles to native disks, combined with differing pH, ionic strength, and lipid compositions, severely hampers structure-activity relation (SAR) interpretation and cross-study comparability.

Compounding these issues is the extensive mutation heterogeneity in rhodopsin-associated retinopathies. Rescue efficacy varies dramatically between variants: some compounds that stabilize P23H may show little to no benefit, or even adverse effects, on other mutants such as T17M, G90V, or C110Y. Deep mutational scanning suggests that less than half of destabilizing mutations are responsive to current chemical chaperones, indicating the need for mutation-stratified or combination approaches. Moreover, assay-context mismatches distort both screening outcomes and mechanistic conclusions. Common experimental systems, such as POPC liposomes or detergent micelles, alter the MI⇌MII equilibrium and hydration profile compared to native rod disk membranes. Trace contaminants like divalent metals or SCN^−^ can further bias intermediate lifetimes [95], yet are rarely controlled.

Temporal complexity is another underappreciated barrier. Rhodopsin activation involves rapid transitions on millisecond to hour-long timescales, from early intermediates to Meta II and Meta III states, yet most high-throughput assays fail to capture or optimize for these kinetics. Ligand screens often prioritize binding affinity over kinetic selectivity, missing opportunities to bias photocycle progression in therapeutically meaningful ways. Finally, delivery, pharmacokinetics, and safety remain major bottlenecks. Achieving effective concentrations in photoreceptors is challenging due to limitations in systemic exposure, volume constraints in intravitreal injections, and metabolic liabilities. Long-term safety, including retinal toxicity, immunogenicity (for biologics), and unintended effects on normal phototransduction, is still poorly understood.

The structural toolkit also lags behind therapeutic ambitions. High-resolution structures are available for only a limited set of chemotypes and rarely for clinically relevant RP mutants in their rescued conformations. Many proposed allosteric sites are inferred from docking or molecular dynamics simulations without experimental validation, constraining structure-based optimization. Addressing these structural blind spots will be critical for rational design of next-generation modulators.

### 4.3. Future Prospects and Strategic Roadmap

To advance rhodopsin modulators from preclinical promise to clinical reality, a shift toward environment-aware, multi-state medicinal chemistry is essential. Future design campaigns must evaluate ligand performance across multiple photocycle states (dark, Meta I, Meta II, Meta III, opsin) under carefully controlled conditions that favor each state (specific pH, ion, and lipid profiles). Importantly, kinetic parameters such as Meta II half-life, Meta III decay rate, and retinal release/rebinding kinetics should be elevated to co-primary endpoints alongside traditional affinity or EC_50_ metrics.

A broader array of targetable surfaces beyond the canonical chromophore binding pocket also deserves attention. Structurally validating novel pockets, including ECL2 plug (nanobody epitopes to small macrocycles), cytoplasmic clefts around conserved microswitch motifs such as E/DRY, and NPxxY, dimer or membrane interfaces stabilized by hydrophobic ligands like econazole analogs, and metal/ion co-binding sites (Na^+^/Zn^2+^ sites), can enable safer, more selective modulation, with fewer photochemical liabilities than retinoid mimetics.

Deliberate engineering of kinetic bias represents another powerful therapeutic axis. Rather than merely lowering basal activity, ligands can be tuned to extend Meta II signaling (beneficial when signaling is weak), collapse Meta III (to reduce toxic late intermediates), or accelerate retinal exchange without destabilizing the fold. Molecular dynamics/adaptive sampling plus flash-photolysis or stopped-flow spectroscopy can guide residence-time engineering.

Given the variant-specific nature of many rhodopsinopathies, precision medicine strategies are imperative. Deep-mutational rescue profiling and clustering of biophysical phenotypes can enable stratified assignment of ligand classes. This opens the door to rational combination therapies; for instance, a folding stabilizer for Class 2 mutants paired with an ECL2-targeted macrocycle for loop-disrupted alleles, or a chromophore prodrug administered alongside a dark-state stabilizer to reduce retinoid-induced loading stress.

Solving the delivery challenge will require formulation innovation. Retina-specific depots, such as intravitreal implants, biodegradable microspheres, or nanoparticles tuned to the DHA/PE lipid ratios of native rod disks, could help maintain therapeutic concentrations while minimizing systemic exposure. Ion-buffered excipients and prodrugs activated only in photoreceptor cells offer additional layers of specificity and safety.

## 5. Conclusions

Rhodopsin continues to serve as both a model system for GPCR biology and a critical therapeutic target for inherited retinal diseases, most notably adRP. The collective evidence reviewed here underscores the recurring structural principles that underlie ligand-mediated stabilization of rod opsin: a hydrophobic anchor that occupies the β-ionone pocket or peri-orthosteric clefts, coupled with a strategically placed polar handle that engages Schiff base counterions or conserved hydrogen-bonding networks. These minimal pharmacophore features recur across both retinoid and non-retinoid chemotypes, dictating folding rescue, stabilization of the dark state, and modulation of photocycle kinetics. This study reveals that while retinoid aldehydes such as 9-*cis*-retinal remain the most potent and broadly effective chemical chaperones, their liabilities, including phototoxicity, instability, and off-target nuclear receptor activation, limit translational potential. In contrast, non-retinoid scaffolds such as chromenones, RS-series spiro-butyrones, YC-001, JC3/JC4, and SRD005825 have demonstrated sub-micromolar efficacy, in vivo photoreceptor protection, and improved safety profiles. Allosteric modulators, including flavonoids, econazole derivatives, Ce6, and cholesterol expand the pharmacological landscape by targeting extracellular loops, cytoplasmic clefts, or dimer interfaces. However, these effects are often mutation-specific, context-dependent, or kinetically complex, highlighting the importance of stratified approaches and mechanistic precision.

Across the therapeutic pipeline, three strategic imperatives emerge here. First, ligand design must become state-aware, with systematic evaluation across the dark state, Meta I, Meta II, and Meta III intermediates, incorporating kinetic endpoints such as Meta II lifetime, retinal release rate, and folding efficiency alongside affinity and thermal stabilization metrics. Second, environment-aware medicinal chemistry is essential, as ligand performance is shaped by lipid composition, ionic milieu, and membrane context. Third, precision medicine approaches are required to address the heterogeneity of rhodopsin mutations, pairing chemical chaperones with complementary modulators or delivery strategies optimized for photoreceptor physiology. Together, these insights point toward a credible roadmap for the rational development of rhodopsin-targeted therapies. By integrating structural knowledge, kinetic control, and genotype-specific strategies, it is now feasible to envision small molecule interventions that move beyond proof-of-concept toward clinical translation. The convergence of medicinal chemistry, deep mutational profiling, and innovative delivery systems will be decisive in realizing the promise of rhodopsin ligands as viable treatments for retinal degenerative disorders.

## Figures and Tables

**Table 1:** Number of unique receptor complexes.

Class (Family)	A (Rhodopsin)	B1 (Secretin)	B2 (Adhesion)	C (Glutamate)	D1 (Ste2-Like Fungal Pheromone)	F (Frizzled)	O1 (Fish-Like Odorant)	O2 (Tetrapod Specific Odorant)	T2 (Taste 2)	Total
Receptors *	185	15	9	13	1	7	3	3	2	238
Receptor-ligand	851	97	13	64	1	19	4	3	6	1058
complexes										
Receptor-G protein complexes	170	15	9	5	1	5	3	3	2	213
Active-state										
receptors **	174	15	9	5	1	5	3	3	2	219

* Receptor-orthologues are only counted once. ** Active state is defined as agonist-bound and opened intracellular TM bundle. https://gpcrdb.org/structure/statistics (accessed on 15 June 2025); [4].

**Table 2:** Rod opsin ligands with therapeutic relevance.

	Ligands	Type	Binding Site	Induced Conformation	References
Retinal Analogs (Schiff base forming)	11-*cis* retinal	Inverse Agonist	Orthosteric	Ground state	[34,44]
	9-*cis*-retinal	Inverse Agonist	Orthosteric	Ground state	[45,46]
	11*-cis*-7-ring retinal	Inverse Agonist	Orthosteric	Ground state	[34,47]
	11*-cis*-6-ring retinal	Inverse Agonist	Orthosteric	Ground state	[34,47]
	11*-cis*-9-demethyl-7-ring retinal	Inverse Agonist	Orthosteric	Orthosteric	[34]
	All*-trans*-retinal	Agonist	Orthosteric	Meta II	[48]
	C17-retinal	Inverse/Partial Agonist	Orthosteric	Ground state	[46]
	C11–C13 five-membered ring-fused 9*-cis*-retinal	Inverse/Partial Agonist	Orthosteric	Ground state	[49]
	C12–C14 five-membered ring-fused 9*-cis*-retinal	Inverse Agonist	Orthosteric	Ground state	[49]
	9*-cis*-retinoyl fluoride	Irreversible Inhibitor	Orthosteric	Ground state	[50]
	all*-trans*-retinoyl fluoride	Irreversible Inhibitor	Orthosteric	N/A	[50]
	13*-cis*-retinoyl fluoride	Irreversible Inhibitor	Orthosteric	Ground state	[50]
	C19-retinyl-amine	Irreversible Inhibitor	Orthosteric	Ground state	[51,52]
	11*-cis*-2,6-dimethyl-8-(2,6,6-trimethyl-1-cyclohexen-1-yl)-3,5,7-octatrien-1-amine	Irreversible Inhibitor	Orthosteric	N/A	[51]
	11*-cis*-n-propyl-retinyl-amine	Irreversible Inhibitor	Orthosteric	N/A	[51]
Retinoid Mimics	9*-cis*-retinyl acetate	Inverse/Pro-Agonist	Orthosteric	Ground state	[48,53]
	all*-trans*-retinol	Agonist	Orthosteric	Meta II	[48]
	all*-trans*-retinoic acid	Agonist	Orthosteric	Meta II	[48,50]
	13*-cis*-retinoic acid	Inverse Agonist	Orthosteric	Ground state	[48]
	5b, 5c, 8c, 11a (orthosteric competitors)	Inverse Agonist	Orthosteric	Ground state	[54]
	β-ionone	Partial Agonist	Orthosteric	Meta II	[38,45,46,50]
	Cyclocitral	N/A	Orthosteric	N/A	[45,46]
	11*-cis*-retinol	Agonist	Orthosteric	Meta II	[45,46]
	5d, 7b, 9d, 10a, 11d, 12 (allosteric stabilizers)	Inverse Agonist	Orthosteric	Ground state	[54]
	9*-cis*-retinyl palmitate	Inverse Agonist	Orthosteric	Ground state	[53]
	13*-cis*-5,8-epoxy-retinoic acid	Inverse Agonist	Orthosteric	Ground state	[55]
	TMAm, TMEs, HNEs, TB3Es, TB4Es, TB35Es	Inverse Agonist	Orthosteric	Ground state	[56]
	CF35Es, and bio-isosteric analogs CF35EsB (boronic acid), CF35EsC (carboxylic acid), CF35EsA (carboxamide)	Inverse Agonist	Allosteric	Ground state	[56]
	SRD005825	Inverse Agonist	Orthosteric	Ground state	[57]
Non-retinoid	NSC 45012	Inverse Agonist	Orthosteric	Ground state	[38]
	*cis*-1,3-dimethyl-cyclohexane	Inverse Agonist	Orthosteric	N/A	[38]
	isoquinoline-2(3H)-hexanamide	N/A	Orthosteric	N/A	[58]
	YC-001	Inverse Agonist	Orthosteric	Ground state	[59,60]
	F5257-0462 (Life Chemical ID)	Inverse Agonist	Orthosteric	Ground state	[61]
	Pocket Competitors—compounds 6, 8, 20, 23	Inverse Agonist	Orthosteric	Ground state	[62]
	Allosteric modulators—compounds 1, 4, 7, 10, 22	Inverse Agonist	Orthosteric	Orthosteric	[62]
	JC3 and JC4	Inverse Agonist	Allosteric	Ground state	[32]
	Chromenone (CR5)	Inverse Agonist	Orthosteric	Ground state	[36]
	RS-Series (RS1–RS4 initial hits; medicinal-chemistry derivatives RS06, RS08, RS09, RS11, RS13, RS15, RS16)	N/A	Orthosteric	Ground state	[63]
	Cyanidin 3-rutinoside	Proposed as Activator	Orthosteric	Ground state	[64]
	Cyanidin-3-O-glucoside (C3G)	Inverse Agonist	Allosteric	N/A	[65]
	Quercetin	Inverse Agonist	Orthosteric/Allosteric	Ground state	[66,67]
	Myricetin	Inverse Agonist	Orthosteric	Ground state	[66,67]
	Myricetrin	Inverse Agonist	Orthosteric	Ground state	[67,68]
	Quercetin-3-rhamnoside	Inverse Agonist	Orthosteric		[67]
	Retigabine (Ezogabine)	Activator	Allosteric	Ground state	[68]
	Chlorin e6 (Ce6)	Inverse Agonist	Allosteric	Ground state	[69,70]
	Sodium valproate	N/A	Allosteric	Ground state	[71]
	Nerol (*cis*-Geraniol; (Z)-geraniol); geraniol (*trans*-Geraniol); Citronellol	Activator	Orthosteric	N/A	PDB IDs: 6PEL, 6PGS, 6PH7
	n-Octyl-Beta-D-Glucopyranoside	N/A	Orthosteric	N/A	PDB IDs: 6NWE, 4X1H, 4J4Q, 4PXF
Non-retinoid Lipidic Compounds	DDHA-PC	N/A	Allosteric	Ground state	[72]
	Cholesterol	N/A	Allosteric	Ground state	[73]
	Tauroursodeoxycholic acid (TUDCA)	Activator	Allosteric	Meta II	[74]
Non-retinoid Dimer Modulators	Econazole; Sulconazole; and six derivatives	Inverse Agonist	Orthosteric/Allosteric	Meta III	[75]
	dimer enhancers: #1 F2502-0030; #2 F5103-0385; #3 F5097-2767; 4 F3382-0749	N/A	Orthosteric	N/A	[76]
	dimer disrupters: #5 F1669-0696; #6 F3215-0002; #7 F5897-0190 (lead disrupter); #8 F0834-0928; #9 F2515-3945	N/A	Orthosteric	N/A	[76]

**Table 3:** Small-molecule ligands and their contribution to rescue rod opsin variants.

Ligand	Breadth/Key Metrics	Assay	Why It Stands Out	References
YC-001	642 out of 1260 mis-trafficking variants rescued	Deep-mutational scanning	Broad mutant coverage; light-stable, non-aldehyde	[58,59,60]
9*-cis*-retinal	67 out of 69 Class 2 variants rescued; EC_90_ ~5 µM	Deep-mutational scanning	Gold-standard chemical chaperone; high affinity	[31,44,55]
JC3 and JC4	30 (JC3) and 26 (JC4) out of 123 mutants rescued, K_d_ = 175 nM (JC3), K_d_ = 98.5 nM (JC4)	Deep-mutational scanning, target binding	Broad mutant coverage, light-stable, in vivo efficacy	[32]
Chromenone (CR5)	31 out of 123 variants rescued; K_d_ = 193 nM	Deep-mutational scanning, target binding	Broad mutant coverage, light-stable, in vivo efficacy	[36]
S-RS1/RS2 (spiro-butanone series)	ΔT_m_ up to +9 °C; rescued trafficking of Rho P23H; EC_50_ = 2.4 µM	Pharmaco-trafficking complementation	First crystallographically solved non-retinoid binders	[63]
13-*cis*-5,8-epoxy-retinoic acid	EC_50_ 0.5–4.8 µM for T17M/P23H/E181K rescued	Cell surface immunostaining	Nanomolar affinity; strong rescue of multiple canonical mutants	[55]
SRD005825	Delays degeneration in T17M mice; IC_50_ 17.8–28.6 µM	Cell surface immunostaining; in vivo treatment	Orally bioavailable non-aldehyde retinoid mimic with in vivo efficacy	[57]
Quercetin/Myricetin	ΔT_m_ +5.6 °C; Meta II t½ +91% in G90V-9CR; rescued trafficking of Rho P23H	Cell surface immunostaining	Cheap, safe, dual allosteric/orthosteric binding; moderate breadth	[66,67]
F5257-0462	Rescued membrane trafficking of 11 mutants	Cell surface immunostaining	non-retinoid small-molecule chaperones	[61]
β-Ionone	Rescued Rho P23H membrane trafficking; strong regeneration inhibitor	Cell surface immunostaining	Simple scaffold; moderate, but consistent, chaperone effect	[38,45,46]

**Table 4:** Key control points of rhodopsin structure–function modulation across retinoid analogs.

Control Knob	Typical Chemical Move	Immediate Structural Effect	Cascading Functional Consequences	References
C10 ⇋ C13 steric tension	delete or add a single CH_3_ (9-dm, 13-dm, 10-Me, 13-iPr) *	the β-ionone pocket (loosens/over-crowds Trp265 & Tyr268)	tunes pre-twist at C11=C12 → quantum-yield spans 0.08–0.67; Meta II pK_a_ shifts 7.7 → 4.5; signaling ranges from 0% to 139%	[82,83]
Proton-transfer switch	electronic pulls (14-F) ** or pushes (ring removal)	raises or lowers SB pKa and the Glu113 ↔ Glu134 proton-relay	full agonist → partial agonist conversion or vice-versa	[84]
Pocket length matching	chain-truncate/extend (C17, C22) or bicyclic locks	over- or under-fills the helix-1/7 corridor	chemical agonism without chromophore, or complete loss of binding	[85,86]
Ring rigidity vs. flexibility	cyclohexenyl locks, 6-member and 5-member braces, α-ring	blocks canonical 11*-cis* → all*-trans* transition or redirects strain	photostable inverse agonists or photocyclic pigments that reset thermally	[79]
Early-polyene shortening	7,8-dihydro-retinal, ring-fused 9-*cis*-retinal	off-loads torsion to C10–C13; HOOP bands collapse	retains WT-like photo-isomerization yet blue-shifts λ_max_; useful for brighter optogenetic scaffolds	[87]

* 9-dm: 9-demethyl-retinal; 13-dm: 13-demethyl-retinal; 10-Me: 10-methyl-retinal; 13-iPr: 13-isopropyl-retinal. ** 14-F: 14-fluoro-retinal.

**Table 5:** Promising non-retinoid chemotypes for pharmacological chaperone development.

Chemotype	Why It Matters	Prototype Ligands	Suggested Next Step	References
Chain-truncated aldehydes	Bind without Schiff base formation, rescue Meta II conformation chemically; small and metabolically flexible	C17 aldehyde → Meta II-like opsin, full rhodopsin-kinase phosphorylation at acidic pH	Add polar tail (amide, sulfonamide) to enhance solubility but keep hydrophobic anchor	[85]
β-Ionone analogs	Minimum scaffold that seals the Trp265 pocket; weak agonism avoids phototoxic Meta II	β-Ionone & oxime series	Diversify ring substitutions (F, CF_3_) to modulate residence time	[85]
Fluoro-acyclic retinals	De-rigidified ring eases entry; 14-fluoro-retinal electronically lowers Schiff base pKa, giving Meta I Schiff base that stabilizes helices but is signaling-silent unless acidified	14-fluoro acyclic retinal	Convert aldehyde → alcohol/amide to prevent undesired Schiff-base	[84]

## Abbreviations

The following abbreviations are used in this manuscript:

adRP	Autosomal dominant retinitis pigmentosa
ATP	Adenosine triphosphate
BRET	Bioluminescence resonance energy transfer
cGMP	Cyclic guanosine monophosphate
DHA/PE	Docosahexaenoic acid/Phosphatidylethanolamine
ECL	Extracellular loop
ERG	Electroretinography
FDA	Food and Drugs Administration
GPCR	G protein–coupled receptor
GRK1	Rhodopsin kinase
G_t_	G protein transducin
ICL	Intracellular loop
LCA	Leber Congenital Amaurosis
LRAT	Lecithin retinol acyltransferase
Meta I	Metarhodopsin I
Meta II	Metarhodopsin II
Meta III	Metharodopsin III
ONL	Outer nuclear layer
OS	Outer segments
POPC	1-palmitoyl-2-oleoyl-sn-glycero-3-phosphocholine
PSB	Protonated Schiff base
RP	Retinitis pigmentosa
RPE	Retinal pigmented epithelium
RPE65	Retinyl pigment epithelium-specific protein 65
SAR	Structure-activity relation
TM	Transmembrane
